# Association between praziquantel treatment and cholangiocarcinoma: a hospital-based matched case–control study

**DOI:** 10.1186/s12885-015-1788-6

**Published:** 2015-10-24

**Authors:** Supot Kamsa-ard, Vor Luvira, Ake Pugkhem, Varisara Luvira, Bandit Thinkhamrop, Krittika Suwanrungruang, Vajarabhongsa Bhudhisawasdi

**Affiliations:** 1 Faculty of Public Health, Khon Kaen University, Khon Kaen, 40002 Thailand; 2 Department of Surgery, Faculty of Medicine, Khon Kaen University, Khon Kaen, 40002 Thailand; 3 Department of Community Medicine, Faculty of Medicine, Khon Kaen University, Khon Kaen, 40002 Thailand; 4 Department of Biostatistics and Demography, Faculty of Public Health, Khon Kaen University, Khon Kaen, 40002 Thailand; 5 Cancer Unit, Srinagarind Hospital, Faculty of Medicine, Khon Kaen University, Khon Kaen, 40002 Thailand

**Keywords:** Cholangiocarcinoma, *Opisthorchis Viverrini*, Repeated Praziquantel, Epidemiology

## Abstract

**Background:**

Infection by the liver fluke, *Opisthorchis viverrini*, remains an important public health problem in Thailand and has resulted in the highest prevalence of infection and incidence of subsequent cholangiocarcinoma (CCA) in the world. Praziquantel (PZQ) is the antihelminthic drug of choice for treatment. Previous studies in hamsters showed that repeated infection and PZQ treatment could increase the risk of CCA. However, the few available epidemiology studies in humans have shown unclear evidence of an increased risk of CCA with frequency of PZQ intake. The present study investigated the relationship between the number of repeated PZQ treatments and CCA.

**Methods:**

A hospital-based matched case–control study was conducted. All cases and controls were inpatients of a tertiary hospital in Northeast Thailand. During 2012–2014 a total of 210 incident cases of pathologically diagnosed CCA and 840 control subjects were selected from a hospital inpatient database (four controls per case). The four recruited controls were individually matched with CCA cases by gender, age and date of admission. Data were collected in face-to-face interviews using a standardised pre-tested questionnaire. Multivariable conditional logistic regression was used in the analysis of the data.

**Results:**

The frequencies of PZQ usage among the 210 cases and 840 controls were 48.6 vs. 66.0 for never, 32.9 vs. 24.4 for once, 8.6 vs. 4.9 for twice, and 10.0 % vs. 4.8 % for more than twice, respectively. There was a statistically significant dose–response relationship (*p* < 0.001). Compared with subjects who never used PZQ, those who used the medication once, twice, and more than twice were 1.49, 1.82, and 2.30 times more likely to develop CCA (95 % confidence intervals: 1.02 - 2.20, 0.92 - 3.60, and 1.20 - 4.40). These odds ratios (adjusted ORs) had already been adjusted for the effects of eating raw fish, a family history of cancer, and highest educational attainment. Additional PZQ usage increased the odds of developing CCA by 23.0 % (adjusted OR = 1.23; 95 % CI: 1.07 - 1.43).

**Conclusions:**

The findings show that repeated PZQ treatments are associated with an increased risk of CCA. Paradoxically, this contradicts the common belief that repeated PZQ treatments decrease the risk of CCA. The study also showed a strong association between the number of repeated PZQ treatments and the consumption of raw freshwater fish. This suggests that repeated PZQ treatments may be a surrogate marker of habit of eating raw fish.

**Electronic supplementary material:**

The online version of this article (doi:10.1186/s12885-015-1788-6) contains supplementary material, which is available to authorized users.

## Background

*Opisthorchis viverrini* (*O. viverrini*), known as the Southeast Asian liver fluke, is a food-borne trematode parasite found in tropical countries. In Thailand, liver fluke infection caused by *O. viverrini* is still an important public health problem. The tradition of eating culturally popular dishes involving the use of raw, partially cooked or underfermented cyprinid fish, which may contain the infective stage (metacercariae) of *O. viverrini*, continues to occur in the northeast region. This practice has resulted in the highest prevalence of *O. viverrini* infection and incidence of subsequent cholangiocarcinoma (CCA) in the world [1–3]. CCA is a bile duct cancer, which originates in biliary epithelial cells, and occurs in the intrahepatic and extrahepatic regions of the bile duct, but it does not include malignancies in the gallbladder or the ampulla of Vater [4–6].

The infection is endemic in the Lower Mekong region of SE Asia, which includes Thailand and the Lao People’s Democratic Republic (Laos PDR). In Thailand, as many as 8 million people are infected with the liver fluke *O. viverrini*, and 2 million in Laos PDR. Approximate 80.0 % of all Thai cases occur in the north and northeast regions of Thailand [7, 8]. Reliable data are only rarely available for the prevalence of *O. viverrini* infection and incidence of CCA in other countries of the Lower Mekong region such as Cambodia and Vietnam. In Cambodia, the apparent infection rate of *Opisthorchis spp.* is 4.0 % [9], while in Vietnam the infection by *O. viverrini* has been reported to be endemic in its southern region [10]. The popular northeast Thai habit of eating raw, undercooked or improperly fermented cyprinid freshwater fish puts people at risk of *O. viverrini* infection [2, 3] when the foods consumed are contaminated by the viable metacercariae of the parasite [8].

The prevalence of liver fluke infection caused by *O. viverrini* in Thailand is distributed predominantly in the north and northeast regions where the rates are 19.3 and 15.7 %, respectively [7, 11]. However, there has apparently been a wide geographical regional and local variation. In the 19 northern provinces, infection rates have been reported to vary between (4.6 – 60.8 %) [12], and similar large variations in rates by intestinal parasites (mainly *O. viverrini*) can also occur within a province [13]. In Khon Kaen Province, prevalence rates of *O. viverrini* between 2.0 and 71 % have been recorded in its various provincial districts [14].

For Thai people, the age-standardized rate (ASR) of liver cancer and bile duct cancer is between 67.6 and 94.8 per 100 000 people in males and between 27.3 and 39.4 per 100 000 in females. The most common histological type is CCA, which comprises between 82.0 and 89.0 % of all detected primary liver cancers [15–20]. In Northeast Thailand, it is estimated that 5000 new cases of CCA are diagnosed every year [21]; this means that each year about 5000 deaths are added to the overall burden of chronic liver and bile duct disease [8].

In Thailand, there have been few previous studies investigating risk factors for CCA, but almost all have emphasized the role of *O.viverrini* infection in the subsequent development of CCA. For instance, the areas with a high incidence of CCA have also been shown to have a high prevalence of *O. viverrini* infection, and both epidemiological studies of humans and laboratory experiments in hamsters have shown that *O. viverrini* infections are associated with CCA [22–25]. Other important potential risk factors for CCA have included antibody titre for *O.viverrini*, the number of praziquantel treatments, the consumption of alcohol, and genetic polymorphism. All of these have been shown to have a statistically significant association with CCA [26–28].

Praziquantel (PZQ) provides effective chemotherapy. It has been and remains the drug of choice to treat *O. viverrini* infection [29, 30]. However, rapid re-infection after successful PZQ treatment has been found to occur, and a high rate of almost 90 % re-infection within one year has been recorded in Khon Kaen Province [12, 31, 32].

People enjoy eating raw, undercooked or inadequately fermented freshwater fish and are aware that PZQ is an effective treatment. Hence, after becoming infected and treated, they return to eating cultural dishes of unsafely prepared fish, become re-infected, and again obtain PZQ for treatment, thus perpetuating the cycle [1, 12, 33]. This seems to have contributed to the continued persistence of CCA in the region [34, 35].

Previous studies in hamsters infected with *O.viverrini* have shown that repeated infection and PZQ treatment may increase the risk of CCA. More frequent *O.viverrini* infections can induce the expression of inducible nitric oxide syntheses (iNOS), not only in inflammatory cells, but also in the epithelium of the bile ducts. This can subsequently cause nitrosative and oxidative damage to nucleic acids, and this damage may play a part in the initiation and/or promotion of steps in the development of CCA [36–38]. Extensive use of repeated doses of PZQ may be a specific factor which needs to be evaluated in relation to the use of this drug and the development of neoplasms in humans [39].

However, the few available epidemiology studies in humans have failed to provide clear evidence of an increased risk of CCA with frequency of PZQ intake. The current study was designed to measure the independent association between CCA and various potential risk factors such as the use of PZQ [26]. One of the problems with previous research into this issue has been the inadequacy of case-definition: for example, in the most relevant previous study histological diagnosis was provided for only 28.0 % of CCA cases. This deficiency could lead to misclassification and could distort the odds ratios, resulting in an underestimate of the real problem. The main purpose of the present study was to investigate the relationship between the number of repeated PZQ treatments and CCA.

## Methods

### Study design

We conducted a hospital-based matched case–control study of patients admitted to Srinagarind Hospital in the city of Khon Kaen, Northeast Thailand. This facility is a regional tertiary referral centre and the main teaching hospital for the Faculty of Medicine at Khon Kaen University.

A total of 210 cases were all incident cases with pathologically confirmed CCA. The controls were 840 subjects, who were selected from the hospital inpatient database (four controls per case). The four controls were individually matched with each CCA case by gender, age (within five years) and date of admission (within three months). Age and gender were chosen as matching variables because previous studies have found that both factors were associated with CCA and repeated PZQ treatments [26, 40].

Data were collected from the recruited inpatients by a face-to-face interview with a trained interviewer using a standardised pre-tested questionnaire (the English language version is attached as an Additional file 1). The questionnaire was developed by researchers and validated by specialists in the field of CCA and a pilot-test conducted on 10 cases and controls. Items in the questionnaire were designed to elicit information about potential risk factors and included questions about demographic variables, the ingestion of raw, partially cooked or possibly underfermented fish, a family history of cancer, use of toxic chemicals, alcohol consumption, smoking behavior, and a history of eating local nitrite-containing foods.

### Eligibility criteria and operational definitions

The eligibility criteria for both cases and controls were:

1. The subject had provided signed informed consent to participate in the study.
2. The subject was able to speak and understand Thai, could provide reliable information, and was both well enough and sufficiently intact cognitively to respond to the interview questions. This criterion was determined by the trained interviewer.

### Cases

The following additional eligibility criteria were used to define an individual as a CCA case:

1. The subject was an inpatient, who was an incident case of CCA and had been admitted and diagnosed between September 1, 2012, and July 31, 2014, at Srinagarind Hospital.
2. The CCA was the primary diagnosis and was histologically confirmed by pathologists from the Department of Pathology in the Faculty of Medicine, Khon Kaen University.

### Controls

The following additional eligibility criteria were used to define an individual as a control:

1. The subject was an inpatient, who was admitted to Srinagarind Hospital between September 1, 2012, and July 31, 2014.
2. The subject was selected from the Srinagarind Hospital inpatient database.
3. The subject had no history of hepatic disease, liver cancer, CCA, or any other malignancy. This was determined from medical records by a researcher assistant. The hospital departments from which the controls were recruited were the Departments of Otolaryngology, Ophthalmology, Rehabilitation, and Orthopedics.

**Fig. 1:**
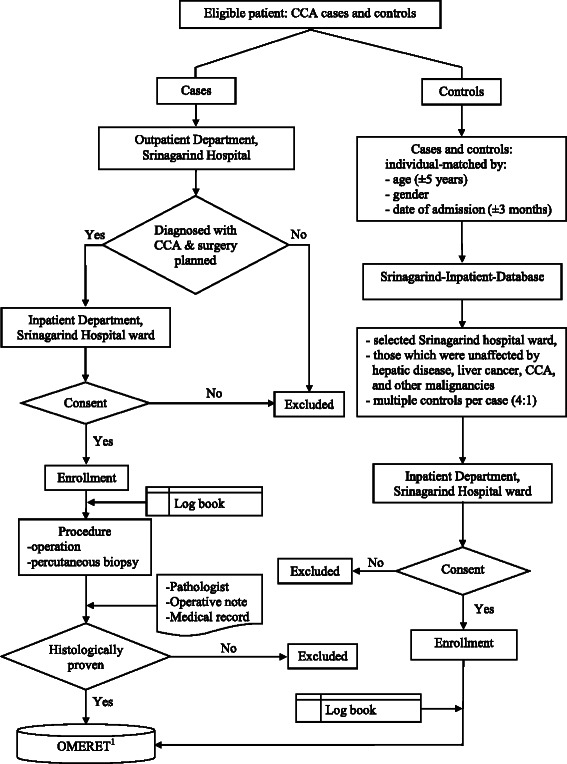
Patient enrollment, cases with CCA and controls

The process for the recruitment and enrolment of cases and controls is summarised in Fig. 1.

### Factor of interest

The factor of interest was the number of repeated treatments with PZQ. The frequency of PZQ treatment was categorised into four groups: never used, used once, used twice, and used more than twice. The trained interviewer showed a sample of PZQ to each participant when asking about PZQ treatment history.

### Potential confounders

Confounding factors were variables which were already known to be associated with CCA. These were various demographic characteristics, use of toxic chemicals, smoking behavior, alcohol consumption, a history of eating local nitrite-containing foods, the consumption of raw, partially cooked, or possibly underfermented fish, and a family history of cancer.

A history of use of toxic chemicals comprised the use of (1) herbicides, (2) rodenticides, (3) insecticides, and (4) fungicides.

Smokers (as opposed to ‘never smokers’) included ex**-**smokers (smoked for at least once day in the past, but quit the habit more than one year previously), occasional smokers (those now smoking less than once per day), and current smokers (those smoking at least once per day).

Drinkers of alcohol (as opposed to ‘never drinkers’) included ex**-**drinkers (used alcohol at least once a day in the past, but quit drinking more than a year previously), occasional drinkers (those presently using alcohol less than once a day), and current drinkers (those using alcohol at least once a day).

A history of eating local nitrite**-**containing foods comprised the eating of (1) salted freshwater fish and salted meat, (2) fermented fish products such as Pla**-**chao and Pla**-**chom, (3) grilled or smoked meat, and (4) sausages (including Chinese sausages).

### Sample size calculation

The estimated required sample sizes for cases and controls were 203 and 812, respectively. This was based on a method of estimating sample sizes in pair**-**matched case control studies [41], an adjustment for the variance inflation factor [42], and the case–control ratio of 1:4 (see an Additional file 2 for details of sample size estimation procedure).

### Data processing

The data were entered into a computer using the Online Medical Research Tools, OMERET [43]. Stata statistical software [44] was used for data verification, and data**-**entry errors were corrected. When questionnaires were found to be incomplete, the participants were contacted by the researcher to obtain the missing information.

### Statistical analyses

#### Description of demographic characteristics of cases and controls

The characteristics of the subjects were summarised using descriptive statistics. Means and standard deviations, medians and their ranges (minimum and maximum) were used for continuous variables, and frequency counts and percentages were used for categorical variables.

### Crude analysis

A crude analysis was performed to determine the associations of the number of repeated PZQ treatments and other factors with CCA without controlling for variable confounding. Crude odds ratios (OR_crude_) and their 95 % confidence intervals (95 % CI) were computed by bivariate conditional logistic regression.

### Test for linear trend

A trend test was used to determine if there was a dose response relationship between the numbers of PZQ treatments and the risk of CCA. The test used was the Mantel**-**Haenszel extension of the chi-square test [41].

### Interaction effects

Possible interaction effects were identified in the bivariate analysis by the use of Mantel**-**Haenszel chi**-**square test of homogeneity [41].

### Multivariable analysis

Multivariable conditional logistic regression [45, 46] was used to compute adjusted odds ratios (OR_adj._) and their 95 % confidence intervals (95 % CI) for investigating the effect of the number of repeated PZQ treatments on CCA, while controlling for the effects of confounding variables. Candidate variables for the multivariable analysis were selected according to two criteria: firstly, variables in the crude analysis which were found to have a p**-**value of less than 0.25, and secondly, variables shown from a literature review to have an association with CCA. The method of backward stepwise elimination was used as the model fitting strategy. A likelihood ratio test was performed to assess the goodness**-**of**-**fit of the final model.

All analyses were performed using Stata version 10.0 [44]. All test statistics were two**-**sided, and a p-value of less than 0.05 was considered statistically significant.

### Ethical considerations

This project was approved by the Human Research and Ethics Committee of Khon Kaen University (Reference No. HE551032). All participants were informed about the objective of the study before being invited to sign the consent form.

## Results

A total of 210 subjects were recruited as CCA cases and 840 were available for analysis as controls. None of the otherwise eligible patients declined consent to participate. Table 1 summarises the outcome of the matching procedure. For all three matching variables, the percentages of cases and control are the same or similar.

**Table 1:** Outcome of case–control matching procedure

Variables	Cases		Controls	
	Number (*n* = 210)	Percent (%)	Number (*n* = 840)	Percent (%)
Gender				
Male	126	60.0	504	60.0
Female	84	40.0	336	40.0
Age at recruitment (years)				
30 **-** 40	3	1.4	14	1.7
41 **-** 50	22	10.5	102	12.1
51 **-** 60	83	39.5	327	38.9
Older than 60	102	48.6	397	47.3
Mean (standard deviation)	60.7 (8.3)		60.3 (8.6)	
Median (minimum: maximum)	60.5 (36.5: 83.2)		60.8 (32.7: 83.2)	
Date of admission (Sep 2012-Jul 2014)				
1^st^ year: recruitment				
Sep 2012 **-** Nov 2012	22	10.5	85	10.1
Dec 2012 **-** Feb 2013	12	5.7	60	7.1
Mar 2013 **-** May 2013	25	11.9	73	8.7
Jun 2013 **-** Aug 2013	28	13.3	107	12.7
2^nd^ year: recruitment				
Sep 2013 **-** Nov 2013	28	13.3	70	8.3
Dec 2013 **-** Feb 2014	54	25.7	199	23.7
Mar 2014 **-** May 2014	21	10.0	139	16.6
Jun 2014 **-** Jul 2014	20	9.5	107	12.7

### Demographic characteristics: unmatched variables

Of the 210 subjects who were recruited as CCA cases, almost all (97.1 %) were born and resided in the northeast (E-san) region, and 92.4 % spoke the local Thai (E**-**san) dialect as their first language. A large proportion (80.5), were married, and 99.5 % were Thai nationals. All reported being Buddhists, 79.1 % had only primary school education, 74.8 % were currently working in agriculture (for example, as farmers or horticulturalists), and their median personal monthly income was 3,000.00 baht (range: 500.00 **-** 90000.00). For the 840 control subjects, the vast majority (92.9) were born and resided in the northeast (E**-**san), and 82.6 % spoke in the local Thai (E**-**san) dialect as their first language. A large proportion (79.5 %), were married, 99.6 % were Thai nationals, 99.6 % were Buddhists, 68.1 % attended only primary schools, 58.7 % currently worked in agriculture, and their median personal monthly income was 40000.00 baht (range: 400.00 **-** 600000.00).

### Crude analysis

The results of this analysis are shown in Table 2. The factors found to be significantly associated with CCA were number of PZQ treatments, highest educational attainment, rodenticide use, alcohol consumption, the eating of raw or partially raw fish (such as Lab**-**Pla, Koi**-**Pla), the eating of fermented fish products (such as Pla**-**chao, Pla**-**chom), the eating of Chinese sausage/other sausage, the eating of fermented fish (Pla**-**ra), and a family history of cancer. Additional PZQ usage increased the odds of developing CCA by 33.0 % (OR_crude_ = 1.33; 95 % CI: 1.16 **-** 1.51; *p* < 0.001). There was a statistically significant dose response relationship (*p* < 0.001).

**Table 2:** Crude odds ratios for CCA associations with various risk factors

Characteristics	Cases (*n* = 210)	Controls (*n* = 840)	Crude OR	95 % CI	*P*-value
Number of PZQ treatments (times)	-	-	1.33	1.16 - 1.51	<0.001
Number of PZQ treatments (times)					<0.001
0	48.5	65.9	1	-	
1	32.9	24.4	1.93	1.35 - 2.77	
2	8.6	4.9	2.72	1.45 - 5.07	
More than 2	10.0	4.8	3.20	1.75 - 5.74	
Test for linear trend (1df)					<0.001
Highest educational attainment					0.003
Primary school	79.1	68.1	1	-	
Secondary school	3.8	6.2	0.51	0.24 - 1.10	
High school or Diploma	9.5	10.5	0.76	0.45 - 1.28	
Bachelor’s degree	7.6	15.2	0.40	0.23 - 0.71	
Herbicide use					0.375
No	79.5	82.0	1	-	
Yes	20.5	18.0	1.20	0.80 - 1.80	
Rodenticide use					0.001
No	70.5	81.1	1	-	
Yes	29.5	18.9	1.81	1.28 - 2.57	
Insecticide use					0.884
No	87.6	87.3	1	-	
Yes	12.4	12.7	0.97	0.60 - 1.56	
Fungicide use					0.421
No	96.7	97.6	1	-	
Yes	3.3	2.4	1.45	0.58 - 3.61	
Smoking behavior					0.045
Never smoked	45.2	50.1	1	-	
Smoker	54.8	49.9	1.74	1.01 - 3.00	
Alcohol use					0.030
Never used	33.8	40.0	1	-	
Drinkers	66.2	60.0	1.60	1.05 - 2.45	
Salted fresh water fish, salted meat					0.474
No	11.0	12.7	1	-	
Yes	89.0	87.3	1.19	0.73 - 1.94	
Raw, partially cooked raw fish (e.g. Lap-Pla, Koi-Pla)					<0.001
No	38.6	61.7	1	-	
Yes	61.4	38.3	3.10	2.18 - 4.40	
Fermented product (e.g. Pla-chao, Pla-chom)					<0.001
No	23.3	35.1	1	-	
Yes	76.7	64.9	1.87	1.30 - 2.71	
Grilled/smoked meat					0.892
No	1.4	1.3	1	-	
Yes	98.6	98.7	0.91	0.25 - 3.36	
Chinese sausage/other sausages					
No	35.2	23.0	1	-	<0.001
Yes	64.8	77.0	0.54	0.38 - 0.75	
Fermented fish (Pla-ra)					0.041
No	1.4	4.6	1	-	
Yes	98.6	95.4	3.5	1.06 - 11.5	
History of cancer in family					<0.001
No	50.0	72.1	1	-	
Yes	50.0	27.9	2.56	1.87 - 3.51	
Combining variables					
Local nitrite-containing foods					0.156
No	57.6	62.9	1		
Yes	42.4	37.1	1.25	0.92 - 1.71	
Environmental chemical use					<0.001
No	55.7	68.7	1		
Yes	44.3	31.3	1.81	1.31 - 2.50	

Herbicide, insecticide and fungicide use, smoking behavior, and the consumption of salted freshwater fish/salted meat, grilled/smoked meat were not significantly associated with CCA.

### Association between CCA, chemical exposures and nitrite-containing food: combining variables

We combined the four chemical variables (herbicide, rodenticide, insecticide, and fungicide use) into one variable, ‘yes’ or ‘no’, depending on whether the subject reported ever having used one or more of the chemicals on at least one occasion. Likewise, we also combined variables about the consumption of local nitrite-containing foods (salted freshwater fish and salted meat, fermented fish products such as Pla-chao and Pla**-**chom, grilled/smoked meat, and Chinese sausage/other sausages) into one variable, ‘yes’ or ‘no’, depending on whether the subject reported ever having consumed one or more of the foods on at least one occasion.

The decision to combine these variables was made because of concern that subjects may have been unable to make reliable distinctions between the four different types of chemical exposures and between the four kinds of nitrite**-**containing foods.

The outcome of combining these variables in the univariate analysis is shown in Table 2.

### Association between use of PZQ treatments and the consumption of raw freshwater fish

There was a statistically significant association between number of PZQ treatments and the consumption of raw freshwater fish (*p* < 0.001) (Table 3).

**Table 3:** Adjusted odds ratios for consumption of raw freshwater fish and number of PZQ treatments

Characteristics	Consuming raw freshwater fish (*n* = 451)	Non-consuming raw freshwater fish (*n* = 599)	Crude OR	Adjusted OR	95 % CI	*P*-value
Number of used PZQ treatments (times)						<0.001
0	54.1	68.8	1	1	**-**	
1	29.0	23.9	1.87	1.87	1.30 **-** 2.70	
2	8.0	3.8	3.19	2.99	1.48 **-** 6.03	
Greater than 2	8.9	3.5	3.27	3.11	1.61 **-** 6.02	

(After adjustment for alcohol consumption and highest educational attainment)

### Interaction term between number of repeated PZQ treatments and chemical environment exposure

A highly significant interaction effect (*p* < 0.001) was found between repeated PZQ treatments and the use of toxic chemicals. The association between CCA and number of repeat PZQ treatments was significantly higher in those reporting use of a toxic chemical.

### Multivariable analysis

#### Factors associated with CCA

Table 4 shows the outcome of the multivariable analysis. The number of PZQ treatments was significantly associated with CCA. Using subjects who never used PZQ as the reference group, the odds of developing CCA for those who used PZQ once, twice, and more than twice were, respectively, 1.49 (95 % CI: 1.02 **-** 2.20), 1.82 (95 CI: 0.92 **-** 3.60, and 2.30 (95 % CI: 1.20 **-** 4.40) times more likely to develop CCA than those who had never used the medication. An additional PZQ use increased the odds of developing CCA by 23.0 % (OR_adj._ = 1.23; 95 % CI: 1.07 **-** 1.43). Subjects who consumed raw freshwater fish had 2.73 times more likely to develop CCA compared with subjects who did not consume this food (OR_adj._ = 2.73; 95 % CI: 1.88 **-** 3.95). A family history of cancer was also a statistically significant risk factor (OR_adj._ = 2.54; 95 % CI: 1.82 **-** 3.95). Education beyond high school/diploma level was a significant protective factor (OR_adj._ = 0.40; 95 % CI: 0.23 **-** 0.71).

**Table 4:** Adjusted odds ratios for CCA associations with various risk factors

Characteristics	Cases (*n* = 210)	Controls (*n* = 840)	Crude OR	Adjusted OR	95 % CI	*P*-value
Number of used PZQ treatments (times)	**-**	**-**	1.33	1.23	1.07 - 1.43	0.005
Number of used PZQ treatments (times)						0.025
0	48.5	65.9	1	1	**-**	
1	32.9	24.4	1.93	1.49	1.02 - 2.20	
2	8.6	4.9	2.72	1.82	0.92 - 3.60	
More than 2	10.0	4.8	3.17	2.30	1.20 - 4.40	
Eating raw fish						<0.001
No	38.6	61.7	1	1	**-**	
Yes	61.4	38.3	3.10	2.73	1.88 - 3.95	
History of cancer in family						<0.001
No	50.0	72.1	1	1	**-**	
Yes	50.0	27.9	2.56	2.54	1.82 **-** 3.55	
Highest educational attainment						0.011
Primary school	79.1	68.1	1	1	**-**	
Secondary school	3.8	6.2	0.51	0.56	0.25 **-** 1.25	
High school/diploma	9.5	10.5	0.76	0.76	0.44 **-** 1.34	
Bachelor’s degree	7.6	15.2	0.40	0.41	0.23 **-** 0.75	

## Discussion

The primary aim of this study was to investigate the relationship between the number of repeated PZQ treatments and CCA. The findings showed that patients who had repeated PZQ treatments were at a substantial risk for developing CCA. This does not mean that there is a direct association between repeated use of PZQ and CCA risk: PZQ is usually only administered when someone is found or suspected to be infected by *O. viverrini,* so that repeat treatments are therefore likely to be confounded with re**-**infection rates.

Other factors found to be highly significantly associated with CCA were the consumption of raw fish, a history of cancer in the family, and educational background.

In Thailand, there is considerable concern about the high incidence of CCA, and previous studies have provided only very limited information about the number of repeated PZQ treatments and other potential risk factors for the disease. Consequently, while we can be highly confident about the role of *O. viverrini* infection, no definitive statements can be made at this time about various other suspected risk factors.

### Risk factors assessment

#### Number of repeated PZQ treatments

For this study, the number of repeated PZQ treatments was significantly and positively associated with an increased risk of CCA, and there was a statistically significant dose response relationship. After its first use, each additional PZQ treatment increased the odds of developing CCA by 23.0 %. This is consistent with a previous study in Thailand [26] which reported statistically significant adjusted odds ratios of 3.04 and 4.60 for those who used PZQ once and more than once. In one study, the odds of developing CCA was found to be 2.16 times more likely to develop CCA in those who used PZQ than in those who had not, but this result was not statistically significant [27]. Similar non**-**significant findings were reported in two other studies [40, 47].

CCA may be induced by the combination of *O. viverrini* infection and exposure to carcinogens such as nitrosamine-rich foods [48, 49]. The proposed pathways linking CCA initiation to the parasite are (1) mechanical damage, (2) molecular products, (3) malfunction and hijacking of the immune system (when combined, these mechanisms result in several common elements which lead to DNA damage), and (4) inhibition of the normal DNA damage response which dramatically increases the likelihood of a malignant transformation [3]. The results of the present study suggest an association between the number of PZQ treatments and the risk of CCA. While repeated PZQ treatments cannot be directly related to CCA, they may be having an effect in combination with *O. viverrini* infections, possibly by initiating or augmenting the mechanisms of opisthorchis**-**derived CCA.

One epidemiological study revealed that subjects with a history of using PZQ on 2–4 occasions were 4.6 times more likely to develop CCA than who never used this drug [48]. This finding might be explained by reference to studies using an animal model. In an experiment with hamsters subjected to a single infection or two or three re-infections, oxidative and nitrosative DNA damage was found occur to faster with successive infections [36]. In another study the use of PZQ as a short-term term treatment of infected hamsters was found to activate an *O.viverrini* antigen burst which induced oxidative and nitrosative stress [50]. To the best of our knowledge, a chronic inflammatory process is one predisposing factor for CCA.

### Number of repeated PZQ treatments and eating raw fish

Importantly, our results also showed that the number of repeated PZQ treatments was significantly associated with the consumption of raw freshwater fish. People who reported repeated PZQ treatments were more likely to report this dietary practice. When people consume raw freshwater fish, they often obtain PZQ as prophylactic treatment in order to eliminate the possibility of consequent *O. viverrini* infection. Paradoxically, many people believe that PZQ has a protective effect against further infection by *O. viverrini* and hence reduces the risk of CCA [51]. PZQ treatment may therefore be a surrogate variable for the behavior of consuming raw freshwater fish and by extension, when not used prophylactically, for infestation with the liver fluke *O. viverrini*.

In the primary prevention of CCA, considerable importance should be attached to reducing the risk of *O. viverrini* infection by attempts to eliminate the practice of consuming raw freshwater fish or freshwater fish products which are unsafely prepared due to undercooking or underfermentation.

### Eating raw fish and CCA risk

Previous studies have shown the eating of raw fish is associated with an increased risk of CCA in Thailand. In one Thai study the odds of developing CCA were 2.94 times more likely to develop CCA in those who ate freshwater fish twice or more per month than in those who had not consumed raw fresh water [27]. Another previous study reported the odds of developing CCA were 1.60, 2.50, and 10.20 times more likely in those who consumed raw freshwater fish monthly, weekly, and daily, respectively, compared with those did not eat raw freshwater fish [13]. Similarly, a further study reported that the odds of CCA were 3.08 and 3.40 times more likely in those who ate raw freshwater fish for 1–4 times per month or three times per week compared with those did not eat raw freshwater fish [47]. All these results are consistent with those obtained in the current study.

The problem of food safety has been shown to be associated with an increased risk of CCA [7]. The traditional habit of eating ground, raw or undercooked or underfermented freshwater fish on a regular basis has repeatedly exposed the local population to liver fluke infection since childhood [52, 53].

National public health control programmes to prevent liver fluke infection caused by *O. viverrini* rely on stool examinations and the treatment of positive cases with PZQ, health education, and the improvement of hygienic defecation [54], but the success of these primary prevention strategies has yet to be convincingly demonstrated [55].

### History of cancer in family

A family history of cancer has previously been shown to be significantly associated with an increased risk of CCA in several previous studies in Thailand. In one study [26], the odds of CCA were 2.10 times more likely to develop CCA in people with a family of history of cancer than in those who reported no family history. In another study [47], patients with a family history of cancer compared with those who did not were found to have 4.3 times the odds of developing CCA: this is higher than the odds found in the present study. In a third study, a positive association was found between a family history of cancer and CCA, but it was not statistically significant [40].

### Highest educational attainment

The finding of the present study that high education to at least a bachelor’s degree level was protective factor for CCA has not previously been reported in a Thai population. One previous case–control study in Northeast Thailand found no association between educational background and CCA, but the subjects were assessed simply as educated or not educated and literate or illiterate [27]. In another northeast Thai case–control study there was similarly no relationship between education and the risk of CCA, but subjects were categorised only as illiterate, completing primary school education and attaining secondary school or higher education, and the proportions of cases and controls receiving secondary school education and beyond were considerably lower than in the present study [13].

One very possible explanation is that the more highly educated people are, the more socially mobile they become. They are therefore more likely to have moved away from their families of origin where there is still a strong cultural pressure to consume traditional dishes containing unsafely prepared fish. In a sense, they have broken free of family pressure to indulge in the strongly ingrained cultural habit of eating raw fish. Of course, such speculations need to be explored in future studies. To the extent that this kind of explanation is correct, it reinforces the importance of strategies designed to break down the cultural attachment to the tradition of eating raw fish. One such strategy is a ‘bottom-up’ approach of working with communities in a participatory way to loosen the cultural bonds with traditional dietary practices [56].

### Strategies to minimise potential biases

#### Histological considerations

A histologically confirmed diagnosis should be required for most cancer patient research participants [57], but almost all studies in Thailand have failed to do this. In the study by Chernrungroj [26] histological confirmation of CCA was obtained for 28.0 % of the cases, in Honjo et al. [27] only 7.0 % of the case diagnoses were histologically confirmed, and similarly the percentage was 7.4 % in Poomphakwaen et al. [40]. Primary outcome and its measurement without histological evidence can lead to cases being incorrectly diagnosed with the result that real exposure differences between cases and controls are diluted, and the chances of demonstrating an effect are reduced. .

### Disease Misclassification

If the controls subsequently become CCA cases, this would introduce a misclassification bias. To minimise the potential for this kind of bias, thorough reviews of medical records, operative notes and especially the surgical pathology reports were obtained in order to confirm that the control group was unaffected by CCA. In reality, such precautions may not always have been successful because CCA is a non-acute disease.

### Information bias

We minimised information bias by ‘blinding’ the trained interviewer and pathologist so that they were unaware whether subjects were in the case or control group. Furthermore, the pathologist did not know the main factor under investigation, and neither did the trained interviewer.

### Confounding bias

The aim of matching was to control for confounder variables such as gender, age (within five years), and date of admission (within three months). We took account of other potential confounding variables by the use of multivariable conditional logistic regression.

### Strengths of the study

There were four important strengths of this study. Firstly, the 210 cases were all incident events diagnosed pathologically of having CCA by a pathologist in the Department of Pathology at the Faculty of Medicine, Khon Kaen University. Secondly, the current study is a hospital-based matched case–control study which had the largest number of pathologically proven CCA subjects examined to date (210) and also the largest number of controls. Thirdly, it is the first published study to investigate the relationship between the number of repeated PZQ treatments and CCA in human subjects. Fourthly, the study demonstrated a dose response relationship between the number of repeated PZQ treatments and CCA risk, and this can be considered as good evidence of an underlying causal relationship [58].

### Limitations of the study

Disease misclassification was possible because some control subjects may have been subsequently diagnosed with CCA. CCA is usually a slow-growing tumor that spreads locally via the lymphatic system, and it is often clinically ‘silent’ until a late stage.

## Conclusions

The findings show that repeated PZQ treatments are associated with an increased risk of CCA. Paradoxically, this contradicts the common belief that repeated PZQ treatments decrease the risk of CCA*.* This study also showed a strong association between the number of repeated PZQ treatments and the consumption of raw freshwater fish, which suggests that repeated PZQ treatments may be a surrogate marker of habit of eating raw fish.

## Additional file

Additional file 1: **English language version of the standardised pre-tested questionnaire.** (PDF 200 kb)
Additional file 2: **Sample size calculation.** (PDF 43 kb)

## Abbreviations

ASR: Age-standardized rate
PZQ: Praziquantel
CCA: Cholangiocarcinoma
*O. viverrini*: *Opisthorchis viverrini*
*Opisthorchis spp.*: *Opisthorchis tenuicollis Opisthorchis felineus Opisthorchis viverrini*
OMERET: Online medical research tools
ORcrude: Crude odds ratios
ORadj.: Adjusted odds ratios
95 % CI: 95 % confidence interval.
