# Supplementary material for: Association between praziquantel treatment and cholangiocarcinoma: a hospital-based matched case–control study
Source: BMC Cancer. 2015 Oct 24;15:776. doi: 10.1186/s12885-015-1788-6 (PMC4619535; doi:10.1186/s12885-015-1788-6)
Supplement: Additional file 1: — English language version of the standardised pre-tested questionnaire. (PDF 200 kb) [file 12885_2015_1788_MOESM1_ESM.pdf]

Sample size calculation

Sample size for pair-matched studies, the total number of pairs: $M \cong m/(p_0q_1 + p_1q_0)$ [41],

where $m = \frac{[Z_\alpha/2 + Z_\beta\sqrt{P(1-P)}]^2}{(P-\frac{1}{2})^2}$; where m represents the number of exposure discordant pairs (b+c); $P = OR/(1+OR)$; p_0 and p_1 represent the proportion of the exposure factor in control and case groups, respectively; $p_1 = p_0OR/[1+p_0(OR-1)]$; $q_0 = 1-p_0$; $q_1 = 1-p_1$. According to previous finding, we assumed p_0 =15%, OR=2.16 [27]. We assumed a level of significance of α = 0.05 ($Z_{\alpha/2} = 1.96$) and the power of the test as β = 0.1 for 90% power ($Z_\beta = 1.28$). The corresponding sample size per group based on a 1:1 ratio was 272 subjects (i.e. Cases=272 and Controls =272). The estimated required sample size was 544 subjects.

The present study was based on multiple controls per case with a ratio between cases and controls of 1:4 resulting in a sample size of cases 170 subjects and controls 680 subjects. The estimated required sample size was 850 subjects as a crude approximation. After adjusting the variance inflation factor (VIF) [42] the partial correlation coefficient = 0.4. The numbers of cases were 203 subjects, and controls were 812. The total sample size was 1015 as a multiple model.

For this present study we collected data of 210 cases and 840 control subjects, so the total sample size was 1050 as is appropriate for an analysis.
