# Supplementary material for: Association between praziquantel treatment and cholangiocarcinoma: a hospital-based matched case–control study
Source: BMC Cancer. 2015 Oct 24;15:776. doi: 10.1186/s12885-015-1788-6 (PMC4619535; doi:10.1186/s12885-015-1788-6)
Supplement: Additional file 2: — Sample size calculation. (PDF 43 kb) [file 12885_2015_1788_MOESM2_ESM.pdf]

Association between praziquantel treatment and cholangiocarcinoma: a hospital-based matched case-control study

Study No: □□□□ **ID-M:** □□□□

ID-Matched: CO □□□□ to CO □□□□ (Please number)

**Age :** □□ years (± 5 years)	**Gender:** □ 1. Male □ 2. Female
Date of admission (IPD): □□/□□/25 □□ (± 3 months)	

Instruction: *Please mark* ☒ *in the box you wish to answer. If the question asks for a number, please write the number in the box* 1 2 3 4 5 6 7 8 9 0, *large enough but not touching the box and one number per box.* ***Please use either black or blue pen***.

Part 1 History of liver fluke infestation, taking praziquantel and other antihelminthic drugs, and chemical exposure

1.1 Question for those who have had stool examination and Opisthorchis viverrini (OV) eggs were found

1.1.1 Have you ever had **"a stool examination"** and **"found OV. eggs"**?

□ 1. Never (If this is the answer, skip to 1.2 on page 2)

□ 2. I have had a stool examination and found OV. eggs

□ 3. I have had a stool examination but OV. eggs were not found
(If other parasites were found, please specify ………………) (Skip to 1.2 on page 2)

□ 9. Not Know/Not sure (Skip to 1.2 on page 2)

1.1.2 If you have had **"a stool examination"** and **"found OV. eggs"**, did you take **"Praziquantel"**? *(Show the sample of praziquantel tablets and its packing to participate before answering the question)*

□ 1. No, praziquantel were taken (Skip to 1.3 on page 2)

□ 2. Yes, praziquantel were taken

□ 9. Don't know/Not sure

1.1.3 How many times did you take **"Praziquantel"**?

□□ times (Please write the number of time, if not know give estimate)

1.1.4 How old were you the first time you took **"Praziquantel"**?

Age □□ years (Please write the number)

1.1.5 How long age was the last time you took **"Praziquantel"**?

□ 1. Days ➔ □□ days   □ 2. Months ➔ □□ months

□ 3. Years ➔ □□ years   □ 9. Not know/Not sure

1.1.6 Normally each time you took praziquantel drugs was when you had **"a stool examination"** and **"found OV. eggs "**

□ 1. Yes   □ 2. No   □ 9. Don't know/Not sure

1.1.7 Where did you get "**the Praziquantel"** from?

□ 1. Self-bought   □ 2. Local health authority   □ 9. From other places, please specify………………..

1.1.8 In one day, how many "**Praziquantel"** tablets do you take?

□□ tablets/day (Please specify the number of tablets)

Study No: □□□□ ID-M: □□□□

1.2 Question for those who "have never had stool examination BUT have taken praziquantel drugs"

1.2.1 Have you ever taken **"Praziquantel"** **"without stool examination"** ?

□ 1. Never (Skip to1.3) □ 2. Yes □ 9. Don't know/Not sure

1.2.2 Within one day how many tablets of "**Praziquantel**" do you take?

□□ tablets/day (Please specify the number of tablets)

1.2.3 How many times have you taken **"Praziquantel"**?

□□ times (Please specify the number of times, if not know, estimate)

1.2.4 When was the last time did you **"take" "Praziquantel"**?

□ 1. Days (Specify number) □□□ days

□ 2. Months (Specify number) □□ months

□ 3. Years (Specify number) □□ years

□ 9. Don't know/Not sure

1.3 Taking other antihelminthic drugs

1.3.1 Have you ever taken antihelminthic drugs**?**

**Antihelminthic drugs (Please specify e.g. trademark, brands of antihelminthic drugs)**		**Number of times (Please specify)**	**Frequency of taking**				**How long ago? (Please specify)**
			**per day (1)**	**per week (2)**	**per month (3)**	**per year (4)**	
………………………… ………………………… …………………………	□ 1. Yes	□□	□	□	□	□	………………… ………………… …………………
………………………… ………………………… ………………………… ………………………… …………………………	□ 2. No						

1.4 Chemical exposure

1.4.1 Have you ever done agricultural work such as farming **excluding** fishery, forestry or livestock?

□ 1. Yes

□ 2. No

Study No: □□□□ ID-M: □□□□

1.4.2 Have you ever used herbicide?

Used of herbicide (Please specify e.g. trademark, brands of herbicide)		Number of times (Please specify)	Frequency of using herbicide				How long have you used herbicide? (Please specify)
			per day (1)	per week (2)	per month (3)	per year (4)	
…………………...<br>…………………...<br>…………………...	□ 1. Yes<br>□ 2. No	□□	□	□	□	□	…………………...<br>…………………...<br>…………………...

1.4.3 Have you ever used rodenticide?

Used of rodenticide (Please specify e.g. trademark, brands of rodenticide)		Number of times (Please specify)	Frequency of using rodenticide				How long have you used rodenticide? (Please specify)
			per day (1)	per week (2)	per month (3)	per year (4)	
…………………<br>…………………<br>…………………<br>…………………	□ 1. Yes<br>□ 2. No	□□	□	□	□	□	……………...…...<br>……………...……...<br>…………...………...<br>…………...………...

1.4.4 Have you ever used pesticide?

Used of pesticide (Please specify e.g. trademark, brands of insecticide)		Number of times (Please specify)	Frequency of using pesticide				How long have you used pesticide? (Please specify)
			per day (1)	per week (2)	per month (3)	per year (4)	
………………<br>………………	□ 1. Yes<br>□ 2. No	□□	□	□	□	□	…………...………...<br>…………...………...

1.4.5 Have you ever used fungicide?

Used of fungicide (Please specify e.g. trademark, brands of fungicide)		Number of times (Please specify)	Frequency of using fungicide				How long have you used fungicide? (Please specify)
			per day (1)	per week (2)	per month (3)	per year (4)	
………………<br>………………<br>………………	□ 1. Yes<br>□ 2. No	□□	□	□	□	□	………...…………<br>………...…………<br>………...…………

Study No: □□□□ ID-M: □□□□

Part 2 General Information

2.1 Height □□□.□ cm Weight □□.□ kg

2.2 What was your weight 5 years ago? □□.□ kg □ 9. Can't remember/Don't know

2.3 Comparing you present weight now and 5 years ago.

□ 1. Increased □ 2. Decreased □ 3. The same

2.4 Marital Status

□ 1. Single □ 2. Married □ 3. Widowed □ 4. Separated

2.5 Nationality

□ 1. Thai □ 2. Chinese □ 3. Hill tribe people (Please specify)……………

□ 4. Others (Please specify)………………..

2.6 Religion

□ 1. Buddhist □ 2. Christian □ 3. Moslem □ 4. Others (Please specify)……..

2.7 Workplace (Please specify province)………………………….... Postal code □□□□□

2.8 Present address (Please specify province)…………………….…. Postal code □□□□□

□ 1. In town □ 2. Suburb

2.9 Place of birth (Please specify province)……………………….... Postal code □□□□□

2.10 Native language

□ 1. Dialect (E-san) □ 2. Thai □ 3. Chinese □ 4. Others (Please specify)…..…

Part 3 Education and Occupation

3.1 Have you ever attended school?

□ 1. Yes □ 2. No (Skip to 3.3)

3.2 Highest level of education

□ 1. Primary school □ 2. Secondary school □ 3. High school

□ 4. Diploma □ 5. University □ 6. Others (Please specify)…………

3.3 Current occupation

□ 1. No job □ 2. Merchant □ 3. Factory worker

□ 4. Laborer □ 5. Government official/State enterprise □ 6. Employee

□ 7. Farmer □ 8. Others (Please specify)...…………………………

3.4 What was your longest occupation? (Please specify)…………………….Duration (Please specify)…………. □□ years

3.5 What is your current income per month (Only your income)? □□□□□□ THB/month

Study No: □□□□ ID-M: □□□□

Part 4 Smoking history

4.1 Have you ever smoked in your life? (Please choose only one answer)

Smoking History	Meaning	Please specify number of cigarettes smoked per (Day/Month/Year)	Starting age	Stopping age
□ 1. Used to	At least once a day and stopped more than a year	□□ /…………	□□	□□
□ 2. Sometimes	Less than once a day	□□ /…………	□□	□□
□ 3. Always	At least once a day	□□ /…………	□□	□□
□ 4. No				

4.2 If you did "**smoke" or "smoke always**", what was your brand? (Choose one option)

□ 1. Filtered government cigarettes □ 2. Non-filtered government cigarettes □ 3. Self-rolled

□ 4. Tobacco chewing □ 5. Others (Please specify)…………………….

4.3 Do you still smoke now?

□ 1. Yes→number of cigarettes □□ (Please specify)

per □ 1. Day □ 2. Month □ 3. Year

□ 2. Stopped at age □□ years

□ 9. Don't remember

Part 5 Alcohol history

5.1 Have your ever drunk alcohol in your life? Such as alcohol, rice whisky, beer, wine or local liquor

□ 1. Yes □ 2. No (Skip to Part 6 number 6.1)

5.2 If you have drunk alcohol before, what is your preferred drink? (Please specify)……………………..

5.3 From 5.2 please give details

Do you still drink alcohol?	Amount per day	Unit per Day	Frequency	How long have you drunk alcohol? (Please specify)
□ 1. Yes	□□ (Please specify)	□ 1. Glass but not flask<br>□ 2. Flask<br>□ 3. Large bottle<br>□ 4. Others (Please specify)…………………..	□ 1. Less than once a month<br>□ 2. 1-3 times /month<br>□ 3. 1 time /week<br>□ 4. 2-5 times /week<br>□ 5. Daily	□□ years
□ 2. No	*If this is your answer please specify age of quitting. If you cannot remember, give an estimate* □□ *years*			

Study No: □□□□ ID-M: □□□□

Part 6 Betel nut chewing history

6.1 Have you ever chewed betel nuts?

☐ 1. Yes

☐ 2. No (Skip Part 7 number 7.1 page 6)

6.2 If you have chewed betel nuts before, please give the following details:

**Do you still chew betel nuts**	**Amount chewed per time**	**Betel nut unit**	**Frequency of chewing**	**How long have you chewed**	**Quitting age**
☐ 1. Yes	□□ (Please specify)	☐ 1. Day<br>☐ 2. Month<br>☐ 3. Year<br>☐ 4. Other (Please specify)…..<br>……………………<br>……………………	☐ 1. Less than once a month<br>☐ 2. 1-3 time/month<br>☐ 3. 1 time/week<br>☐ 4. 2-5 time/week<br>☐ 5. Daily	□□ years	…………..
☐ 2. No	*If this is your answer, please specify the age you stopped or estimate* □□ *years*				

Part 7 History of residential environment

7.1 Last year, where was the toilet?

☐ 1. In the house ☐ 2. Outside the house/With roof and walls ☐ 3. No toilet

7.2 Last year what was your water source?

☐ 1. Tap water ☐ 2. Wall water

☐ 3. River water ☐ 4. Others (Please specify)……………………………………….

Study No: □□□□ ID-M: □□□□

Part 8 History of eating local nitrite-containing foods and using herbs

8.1 Have you ever eaten the following foods?

Foods		Number of times	Frequency of eating				How many years age? (Please specify)
			per day (1)	per week (2)	per month (3)	per year (4)	
1) Salted fish water fresh, salted meat	□ 1. Yes<br>□ 2. No	□□	□	□	□	□	□□ years
2) Raw fish, Partial raw fish (e.g. Lap-Pla, Koi-Pla)	□ 1. Yes<br>□ 2. No	□□	□	□	□	□	□□ years
3) Fermented product (e.g. Pla-chao, Pla-chom)	□ 1. Yes<br>□ 2. No	□□	□	□	□	□	□□ years
4) Meat grill/ smoked	□ 1. Yes<br>□ 2. No	□□	□	□	□	□	□□ years
5) Chinese sausage/ other sausage	□ 1. Yes<br>□ 2. No	□□	□	□	□	□	□□ years
6) Pickled vegetables or fruits	□ 1. Yes<br>□ 2. No	□□	□	□	□	□	□□ years

8.2 History of herb usage in the last 3 years

Herb usage history		Number of time	Frequency of consumption			
			per day (1)	per week (2)	per month (3)	per year (4)
1) In last 3 years, have your ever used Chinese herbs or Thai herbs to treat illness?	□ 1. Yes<br>□ 2. No	□□	□	□	□	□

2) How long did you use the above mentioned herbs? □□ years □□ months (Please specify the number)

3) Please specify the name of herbs used.

……………………………………………………………………………………………
……………………………………………………………………………………………
……………………………………………………………………………………………
……………………………………………………………………………………………

Study No: □□□□ ID-M: □□□□

Part 9 History of eating fermented fish (Pla-ra)

9.1 Eating fermented fish

**Eating fermented fish**		**Number of times per month (Please specify)**	**Frequency of eating fermented fish**	**How long have you eaten fermented fish?**
1) Have you ever eaten fermented fish?	☐ 1.Yes	□□	☐ 1. Less than once a month<br>☐ 2. 1-3 time/month<br>☐ 3. 1 time/week<br>☐ 4. 2-5 time/week<br>☐ 5. Daily	□□ years
	☐ 2. No	*(Skip to part 10 number 10.1 page 10)*		

9.2 Type of fermented fish (Choose only one)

**Type of fermented fish**		**Number of times per month (Please specify)**	**Frequency of eating fermented fish**	**How long have you eaten fermented fish?**
1) Do you normally eat cooked fermented fish? (Including used for cooking or eating separately)	☐ 1. Yes	□□	☐ 1. Less than once a month<br>☐ 2. 1-3 time/month<br>☐ 3. 1 time/week<br>☐ 4. 2-5 time/week<br>☐ 5. Daily	□□ years
	☐ 2. No			
2) Do you normally eat raw fermented fish? (Including used for cooking or eating separately)	☐ 1. Yes	□□	☐ 1. Less than once a month<br>☐ 2. 1-3 time/month<br>☐ 3. 1 time/week<br>☐ 4. 2-5 time/week<br>☐ 5. Daily	□□ years
	☐ 2. No			

Study No: □□□□ ID-M: □□□□

9.2 Type of fermented fish (choose only one)

**Type of fermented fish**		**Number of times per month (Please specify)**	**Frequency of eating fermented fish**	**How long have you eaten fermented fish?**
3) Do you normally eat half-cooked fermented fish?	□ 1. Yes	□□	□ 1. Less than once a month<br>□ 2. 1-3 time/month<br>□ 3. 1 time/week<br>□ 4. 2-5 time/week<br>□ 5. Daily	□□ years
	□ 2. No			

9.3 Fermented fish source

9.3.1 Where dose the fermented fish you eat come from? (Please choose only one answer)

**Fermented Fish source**		**Amount of fermented fish per time (1 Haisong =30 kgs.)**	**Frequency of producing fermented fish**
1) Where dose the fermented fish you eat come from?	□ 1. Self-made	□□	□ 1. per day<br>□ 2. per week<br>□ 3. per month<br>□ 4. per year
	□ 2. Bought	□□	□ 1. per day<br>□ 2. per week<br>□ 3. per month<br>□ 4. per year
	□ 3. Self-made and bought	□□	□ 1. per day<br>□ 2. per week<br>□ 3. per month<br>□ 4. per year

9.4 If you made the fermented fish, would you finish 1 Haisong (30 kgs.) in 1 year

□ 1. Yes □ 2. No □ 9. Don't know/Not sure

9.5 How many people share the same Haisong of fermented fish? □□ People (Please specify number)

9.6 How many adults? □□ people

9.7 How many children? (Age less than 12 years) □□ people

9.8 Do you still eat fermented fish?

□ 1. Yes, □□ Times per day (Please specify number)

□ 2. No, I stopped at age □□ years

□ 9. Don't remember

Part 10 Health history

10.1 Do you have "**Viral hepatitis**"?

□ 1. Yes □ 2. No (Skip to 10.3) □ 3. Don't know/Never investigated (Skip to 10.3)

10.2 If you have "**Viral hepatitis**" , please give the following detail:

**Viral hepatitis**		**Age Infection started**	**Viral hepatitis treated or non**	**Type of treatment**
1) hepatitis A	□ 1. Yes	□□	□ 1. Yes	……………………..……
	□ 2. No		□ 2. No	
2) hepatitis B	□ 1. Yes	□□	□ 1. Yes	……………………..…
	□ 2. No		□ 2. No	
3) hepatitis C	□ 1. Yes	□□	□ 1. Yes	……………………..…
	□ 2. No		□ 2. No	
4) unknown type of viral hepatitis	□ 1. Yes	□□	□ 1. Yes	……………………..……
	□ 2. No		□ 2. No	
5) others type (Please specify)….. …………………… …………………… ……………………	□ 1. Yes	□□	□ 1. Yes	………………………..
	□ 2. No		□ 2. No	

Study No: ☐☐☐☐ ID-M: ☐☐☐☐

10.3 Have you ever had the following diseases? (Not now)

**Diseases**		**Age of onset (years)**	**Is it recurring**	**Mode of Treatment**
1)Tuberculosis	☐ 1. Yes	☐☐	☐ 1. Yes<br>☐ 2. No<br>☐ 9. Don't know	☐ 1. Modern Medicine<br>☐ 2. Traditional Medicine<br>☐ 3. Others (Please specify)…..
	☐ 2. No			
2) Malaria	☐ 1. Yes	☐☐	☐ 1. Yes<br>☐ 2. No<br>☐ 9. Don't know	☐ 1. Modern Medicine<br>☐ 2. Traditional Medicine<br>☐ 3. Others (Please specify)…..
	☐ 2. No			
3) Cancer	☐ 1. Yes	☐☐	☐ 1. Yes<br>☐ 2. No<br>☐ 9. Don't know	☐ 1. Modern Medicine<br>☐ 2. Traditional Medicine<br>☐ 3. Others (Please specify)…..
	☐ 2. No			
4) Sexually Transmitted Diseases	☐ 1. Yes	☐☐	☐ 1. Yes<br>☐ 2. No<br>☐ 9. Don't know	☐ 1. Modern Medicine<br>☐ 2. Traditional Medicine<br>☐ 3. Others (Please specify)…..
	☐ 2. No			

10.4 Have you ever had **"Diabetes"**

**Diabetes**		**Age of onset (years)**	**Mode of Treatment**
	☐ 1. Yes	☐☐	☐ 1. Insulin injections<br>☐ 2. Oral medication<br>☐ 3. Others (Please specify)………………………
	☐ 2. No		

Study No: ☐☐☐☐ ID-M: ☐☐☐☐

Part 11 History to using hormones and pregnancy (Only for females)
(If male skip to Part 12 number 12.1)

11.1 Usage of contraceptives or hormones

**Hormone usage**	**Objective of Hormone usage**	**Number of times**	**Frequency of using hormones**	**Type of Hormone Treatment**	**How long did you use?**
1) Have you ever taken "contraceptive"?<br>☐ 1. Yes<br>☐ 2. No	☐ 1. Contraception<br>☐ 2. Menopause Treatment<br>☐ 3. Others (Specify)…………	☐☐	☐ 1. Daily<br>☐ 2. Weekly<br>☐ 3. Monthly<br>☐ 4. Annually	☐ 1. Tablet<br>☐ 2. Injection<br>☐ 3. Others (Specify)……<br>………............	………..
2) Have you ever taken other hormones?<br>☐ 1. Yes<br>☐ 2. No	☐ 1. Contraception<br>☐ 2. Menopause Treatment<br>☐ 3. Others (Specify)…………	☐☐	☐ 1. Daily<br>☐ 2. Weekly<br>☐ 3. Monthly<br>☐ 4. Annually	☐ 1. Tablet<br>☐ 2. Injection<br>☐ 3. Others (Specify)……<br>………............	………..

11.2 Pregnancy

**Pregnancy**		**Age of first pregnancy**	**Total number of pregnancy**
1) Have you ever been pregnancy?	☐ 1. Yes	☐☐	☐☐
	☐ 2. No	*Skip to Part 12 number 12.1*	

Part 12 History of receiving blood, donating blood and history of cancer in the family

12.1 **Have you ever received blood, (excluding this time)**

**Blood Transfusion**		**Frequency of "Blood Transfusion"**	**What was the year of your last transfusion?**
1) Have you ever received blood?	☐ 1. Yes<br>☐ 2. No	☐ 1. 1 time<br>☐ 2. 2-4 times<br>☐ 3. 5 more than 5 times<br>☐ 4. Don't know	B.E 25 ☐☐

Study No: ☐☐☐☐ ID-M: ☐☐☐☐

12.2 Have you ever donated blood?

**Blood donated**		**Frequency of donating blood**	**When was the time you donated?**
1) Have you ever donated blood	☐ 1. Yes<br>☐ 2. No	☐ 1. 1 time<br>☐ 2. 2-4 times<br>☐ 3. 5 more than 5 times<br>☐ 4. Don't know	B.E 25 ☐☐

12.3 Have you ever had the following experiences?

**Experience**	**Age of onset** (Please specify)	**Comment (What you want to let doctor know?)**
1) Operation (Excluding this time)<br>☐ 1. Yes ☐ 2. No	☐☐	
2) Tattoo<br>☐ 1. Yes ☐ 2. No	☐☐	
3) Body piercing such as tongue, ear<br>☐ 1. Yes ☐ 2. No	☐☐	
4) Circumcision<br>☐ 1. Yes ☐ 2. No	☐☐	
5) Dental operation such as tooth filling, tooth extractor, root canal treatment<br>☐ 1. Yes ☐ 2. No	☐☐	

Study No: □□□□ ID-M: □□□□

12.4 Family history of cancer

**Family history of cancer**		**Family member that had cancer**	**Location of cancer**
1) Family member with history of cancer (family member includes grandparent, parents, children or grandchildren)	□ 1. Yes	□ 1. Father	
		□ 2. Mother	
		□ 3. Sibling	
		□ 4. Son/Daughter	
		□ 5. Paternal grandfather	
		□ 6. Paternal grandmother	
		□ 7. Father's siblings	
		□ 8. Maternal grandfather	
		□ 9. Maternal grandmother	
		□ 10. Mother's siblings	
		□ 11. Grandchildren	
		□ 12. Others (Please specify)……………	
	□ 2. No		
	□ 9. Don't know/ Not sure		

Date of interview:…………………/………………../ B.E 25………..
Name of interview:………………………………………......... (Print)

Thank you for your time
Research team
